# Effectiveness of the chronic care model for adults with type 2 diabetes in primary care: a systematic review and meta-analysis

**DOI:** 10.1186/s13643-022-02117-w

**Published:** 2022-12-15

**Authors:** Lay Hoon Goh, Chiew Jiat Rosalind Siah, Wilson Wai San Tam, E Shyong Tai, Doris Yee Ling Young

**Affiliations:** 1 Division of Family Medicine, Yong Loo Lin School of Medicine, National University of Singapore, NUHS Tower Block Level 9, 1E Kent Ridge Road, Singapore, 119228 Singapore; 2 Alice Lee Centre for Nursing Studies, Yong Loo Lin School of Medicine, National University of Singapore, Singapore, Singapore; 3 Department of Medicine, Yong Loo Lin School of Medicine, National University of Singapore, Singapore, Singapore

**Keywords:** Chronic Care Model, Chronic disease, Disease management, Meta-analysis, Patient care team, Self-management, Systematic review, Type 2 diabetes

## Abstract

**Background:**

Mixed evidence exists regarding the effectiveness of the Chronic Care Model (CCM) with patient outcomes. The aim of this review is to examine the effectiveness of CCM interventions on hemoglobin A1c (HbA_1c_), systolic BP (SBP), diastolic BP (DBP), LDL cholesterol and body mass index (BMI) among primary care adults with type 2 diabetes.

**Methods:**

PubMed, Embase, CINAHL, Cochrane Central Registry of Controlled Trials, Scopus and Web of Science were searched from January 1990 to June 2021 for randomized controlled trials (RCTs) comparing CCM interventions against usual care among adults with type 2 diabetes mellitus in primary care with HbA_1c_, SBP, DBP, LDL cholesterol and BMI as outcomes. An abbreviated search was performed from 2021 to April 2022. This study followed the Preferred Reporting Items for Systematic reviews and Meta-Analyses (PRISMA) guidelines for data extraction and Cochrane risk of bias assessment. Two reviewers independently extracted the data. Meta-analysis was performed using Review Manager software. Heterogeneity was evaluated using χ^2^ and *I*^2^ test statistics. Overall effects were evaluated using *Z* statistic.

**Results:**

A total of 17 studies involving 16485 patients were identified. Most studies had low risks of bias. Meta-analysis of all 17 studies revealed that CCM interventions significantly decreased HbA_1c_ levels compared to usual care, with a mean difference (MD) of −0.21%, 95% CI −0.30, −0.13; *Z* = 5.07, *p*<0.00001. Larger effects were experienced among adults with baseline HbA_1c_ ≥8% (MD −0.36%, 95% CI −0.51, −0.21; *Z* = 5.05, *p*<0.00001) and when four or more CCM elements were present in the interventions (MD −0.25%, 95% CI −0.35, −0.15; *Z* = 4.85, *p*<0.00001). Interventions with CCM decreased SBP (MD −2.93 mmHg, 95% CI −4.46, −1.40, *Z* = 3.75, *p*=0.0002) and DBP (MD −1.35 mmHg, 95% CI −2.05, −0.65, *Z* = 3.79, *p*=0.0002) compared to usual care but there was no impact on LDL cholesterol levels or BMI.

**Conclusions:**

CCM interventions, compared to usual care, improve glycaemic control among adults with type 2 diabetes in primary care, with greater reductions when the mean baseline HbA_1c_ is ≥8% and with interventions containing four or more CCM elements.

**Systematic review registration:**

PROSPERO  CRD42021273959

**Supplementary Information:**

The online version contains supplementary material available at 10.1186/s13643-022-02117-w.

## Background

Chronic diseases are increasing globally and have a significant impact on primary health services. Diabetes in particular is a complex disease that has considerable complications related to cardiovascular morbidities, thus leading to a poor quality of life [1, 2]. The global diabetes prevalence in adults in 2021 was estimated to be 10.5%, approximately 537 million adults, with the figure rising to 12.2% at 783 million by 2045 [3]. In that year, diabetes caused 6.7 million deaths and caused at least USD 966 billion dollars in health expenditures, with 9% of total spending on adults. People with chronic conditions have multifaceted and complex needs that require continuity, comprehensiveness and coordination, of which primary care can play a central role in effective management and care integration [4]. However, patients often receive inadequate care with limited physician engagement in disease management as well as little coordination and communication among care providers [5].

Integrated care models are found to be effective in reducing health care costs and hospitalizations [6–9], besides enhancing patient satisfaction, increasing perceived quality of care and enabling access to services [10]. The 2022 American Diabetes Association (ADA) Standards of Medical Care in Diabetes [11] recommended that the approach to diabetes management in primary care be aligned with the Chronic Care Model (CCM), which emphasizes person-centred team care, integrated long-term treatment approaches to diabetes and comorbidities, and ongoing collaborative communication and goal setting between all team members. The CCM centred in primary care was developed by Wagner in the 1990s and has been shown to provide the best evidence-based framework for organizing and optimizing diabetes care delivery by modifying essential healthcare system elements to support high-quality patient-centred management [12–14]. These six elements are the organization of the healthcare delivery system, community linkages or resources, self-management support, decision support, delivery system design and clinical information systems and have been used as interventions to show improvement in diabetes care [15–18]. The CCM elements are described as follows, based on a published description [19]: **Organization of healthcare delivery system** refers to a health system’s business plan to create a quality-oriented culture of providing safe and high quality care and reflects its commitment to apply the CCM across the organization. Features of this element include: (i) presence of clinician leaders who are dedicated members of the team and who visibly support improvement at all levels of the organization, beginning with the senior leader, (ii) promoting effective improvement strategies aimed at comprehensive system change, (iii) encouraging open and systematic handling of errors and quality problems to improve care, (iv) providing incentives based on quality of care and (v) developing agreements that facilitate care coordination within and across organizations.

**Community linkage** refers to mobilizing or developing community resources and policies to support healthy lifestyles and the needs of patients. Community resources help bolster health systems efforts to keep chronically ill patients supported, involved and active. Features of this element include: (i) encouraging patients to participate in effective community programmes, (ii) forming partnerships with community organizations to support and develop interventions that fill gaps in needed services and (iii) advocating for policies that improve patient care.

**Self-management support** refers to empowering and preparing patients to manage their health care. Patients are encouraged to set goals, identify barriers and challenges, and monitor their own conditions. A variety of tools and resources provide patients with visual reminders to manage their health. Features of this element include: (i) emphasizing the patient’s central role in managing their health, (ii) using effective self-management support strategies that include assessment, goal setting, action planning, problem-solving and follow-up and (iii) organizing internal and community resources to provide ongoing self- management support to patients.

**Delivery system design** refers to assuring effective, efficient care and self-management support in care delivery. Features of this element include: (i) regular, proactive planned visits which incorporate patient goals to help individuals maintain optimal health and allow health systems to better manage their resources, (ii) visits often employ the skills of several team members with defined roles and tasks, (iii) using planned interactions to support evidence-based care, (iv) providing clinical case management services for complex patients, (v) ensuring regular follow-up by the care team and (vi) giving care that patients understand and that agrees with their cultural background.

**Decision support** refers to promoting care consistent with evidence-based, effective care guidelines and patient preferences. Features of this element include (i) clinicians have convenient access to the latest evidence-based guidelines for care for each chronic condition, (ii) continual educational outreach to clinicians to reinforce utilization of these standards, (iii) embedding evidence-based guidelines into daily clinical practice, (iv) sharing evidence-based guidelines and information with patients to encourage their participation, (v) using proven provider education methods and (vi) integrating specialist expertise and primary care.

**Clinical information systems** refer to organizing data to facilitate efficient and effective care. Features of this element include: (i) health systems that harness technology to provide clinicians with an inclusive list (registry) of patients with a given chronic disease. A registry provides the information necessary to monitor patient health status and reduce complications, (ii) providing timely reminders for providers and patients, (iii) identifying relevant subpopulations for proactive care, (iv) facilitating individual patient care planning, (v) sharing information with patients and providers to coordinate care and (vi) monitoring performance of practice team and care system.

Previous systematic reviews and meta-analyses showed research gaps and mixed results in evaluating CCM interventions in patients with type 2 diabetes for patient outcomes such as hemoglobin A1c (HbA_1c_), systolic blood pressure (SBP), diastolic blood pressure (DBP), low-density lipoprotein (LDL) cholesterol and body mass index (BMI). The HbA_1c_ measurement remains the primary tool for assessing glycemic control and risk for diabetes complications and mortality in medical evaluation [20–23]. Previous meta-analyses of CCM were based on a limited number of search databases and included non-randomized trials, patients with type 1 diabetes or studies limited to a continent [24–30]. Evidence from some systematic reviews showed a mixed impact on patient outcomes and processes of care [31–34], while other meta-analyses showed improvements in patient outcomes such as HbA_1c_ [35–37].

A retrospective cohort study in southern England using databases [38] showed that the rate of utilization of primary care services by people with type 2 diabetes increased from 2013 to 2020, but this increase did not correlate with better outcomes. The World Health Organization’s Global Diabetes Compact [39], a global initiative, proposed strengthening primary health care for accessible diabetes treatment. It is therefore timely to perform an updated systematic review and meta-analysis to enhance applicable knowledge for the management of type 2 diabetes in primary care. Our study will update the literature search up to 2022 that examined CCM interventions compared with usual care and controls using patient outcomes such as HbA_1c_, SBP, DBP, LDL cholesterol level and BMI in patients with type 2 diabetes receiving primary care.

## Methods

This systematic review was conducted according to the Cochrane Handbook for Systematic Reviews of Interventions [40] and reported with reference to the Preferred Reporting Items for Systematic reviews and Meta-Analyses (PRISMA) statement [41]. The protocol of this systematic review was registered in the International Prospective Register of Systematic Reviews (PROSPERO CRD42021273959).

### Literature search

For this review, a comprehensive search of randomized controlled trials (RCTs) from January 1990 (around when CCM was introduced) until 11 June 2021 was performed. Six databases, PubMed, Embase, CINAHL, Cochrane, Scopus and Web of Science, were searched. A two-phase search strategy was used for this review. In the first phase, an initial search of PubMed was performed using the following keywords and Medical Subject Headings (MeSH) terms: *Diabetes Mellitus, Type 2, Models, Theoretical, Disease Management, Patient Care Team, Patient-Centred Care, Patient Care Management, Self Care, Self Efficacy, Delivery of Health Care, Self-Management and Chronic Disease*. The search terms used in this study are shown in Additional file 1. Studies appearing to fit the eligibility criteria were retrieved. From these, relevant keywords and MeSH terms that were used in these studies were identified and compiled for a more thorough search to ensure that relevant studies on the topic were not missed. Through the studies identified in the first phase, a list of relevant keywords and MeSH terms was compiled. This was then used in the second phase, where the six databases, as identified above, were searched from January 1990 until June 2021. A manual search was also performed by searching the reference lists of eligible papers. An abbreviated search update was performed (2021 to 28 April 2022) using the PubMed, Embase and CINAHL databases.

### Inclusion criteria

Studies were included if they met the following criteria: (i) non-pregnant adult patients 18 years old and above with type 2 diabetes receiving care in primary care; (ii) interventions that included CCM elements such as the organization of the healthcare delivery system, community linkages or resources, self-management support, decision support, delivery system design and clinical information systems; (iii) usual care as control; (iv) post-intervention HbA_1c_ level as outcomes; and (v) RCTs. For studies that did not describe the CCM elements within the interventions, two reviewers, LHG and CJRS, did so based on the published description of CCM elements as described [19].

### Exclusion criteria

Studies were excluded if they involved (i) children; (ii) acute diseases, cardiovascular diseases, chronic respiratory diseases, human immunodeficiency virus, mental health disorders, chronic pain and cancer and (iii) hospital or nursing homes. Usual care refers to standard of care for patients with type 2 diabetes. Studies that included additional interventions into their usual care will be excluded.

### Selection of studies

The selection process is reported using a PRISMA flow diagram [41]. The studies identified were exported to EndNote X9.3.3, where duplicate records were removed manually [42]. Two reviewers (LHG and CJRS) independently screened the titles and abstracts against the eligibility criteria and removed irrelevant records. Studies that appeared to fit the above criteria were retrieved in full for further assessment by the two reviewers, and irrelevant records were removed. Publications generated from the same study were linked together. When published information was insufficient to decide whether to exclude or include the study, the authors of these studies were contacted to acquire the necessary information. LHG and CJRS validated the final list of included studies. A third reviewer (WWST) was consulted if disagreements between LHG and CJRS were not resolved through discussion.

### Data extraction

Reviewer LHG extracted and summarized relevant data of included studies using the standardized data extraction sheet according to the Cochrane Handbook for Systematic Reviews of Interventions [43] with details such as the author’s details, year of publication, country, study design, setting, participants’ characteristics, intervention, number and type of CCM elements used, control, sample size, attrition rate, outcomes and number included in the analysis. Both primary and secondary outcomes were extracted. When the outcomes were reported and presented as continuous data, the mean and standard deviation (SD) were extracted for both the control and intervention groups at follow-up. When the studies reported more than one follow-up period, data were only extracted for the latest follow-up from the start of the intervention. Where data were not reported as the mean and SD, such as if the authors reported the results as the median and interquartile range, we contacted the author of the study to request the relevant data. If there was no response from the authors, the quantile method from Wan et al. [44] was used to calculate the mean and SD.

### Quality assessment

The methodological quality of the included studies was assessed independently by two reviewers (LHG and CJRS) using the Cochrane Collaboration’s risk of bias tool [40]. This tool assessed each study for random sequence generation, allocation concealment, blinding of participants and personnel, blinding of outcome assessment, incomplete outcome data and selective reporting. Any disagreements between the two reviewers were resolved through discussion with a third reviewer (WWST). The risk of bias graph and summary were generated by Review Manager (RevMan) software 5.4.1 [45].

### Data synthesis

The primary outcome of this review was HbA_1c_. The secondary outcomes were SBP, DBP, LDL cholesterol and BMI. All outcomes were expressed as the mean differences (MD) with 95% confidence interval (CI). The results were considered statistically significant when *p*<0.05. The results were pooled using DerSimonian and Laird’s random-effects model. RevMan software was used to conduct meta-analyses and graph generation. The heterogeneity of the selected studies was evaluated using χ^2^ and *I*^2^ statistics [46]. Using the χ^2^ test, significant heterogeneity between studies was considered significant if *p*<0.10 [47]. Using *I*^2^ statistics, 0–40% represents no importance, 30–60% moderate heterogeneity, 50–90% substantial heterogeneity and 75–100% considerable heterogeneity [46]. Subgroup analysis explored the effectiveness of CCM across baseline HbA_1c_ levels, study duration and numbers of CCM elements. The baseline HbA_1c_ at 8% was chosen as a less stringent treatment goal in consideration of the heterogeneity of preferences for intensity and mode of glucose control in older adults with type 2 diabetes [48–51]. Publication bias was explored using a funnel plot and Egger’s test [52].

## Results

### Search results

**Fig. 1:**
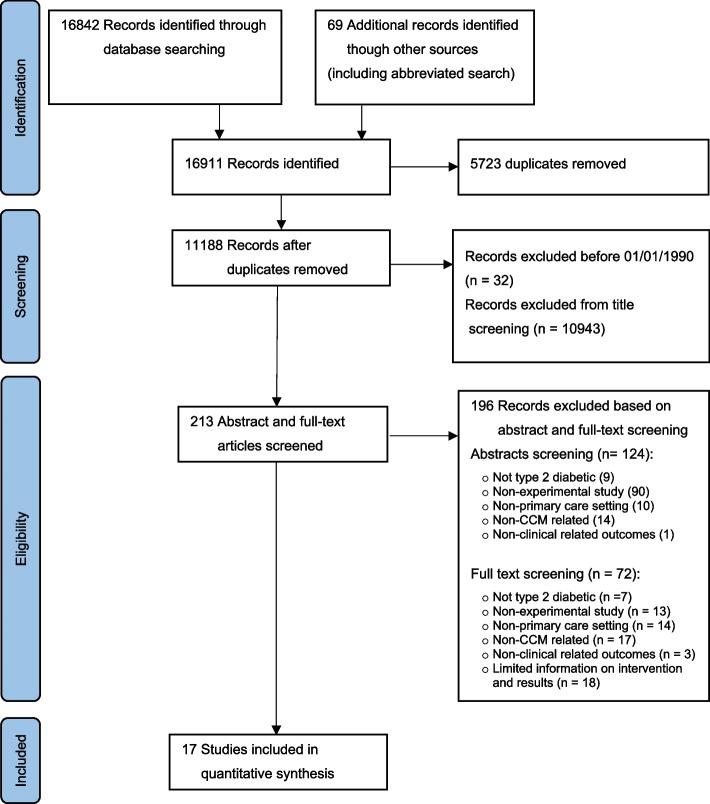
Flow diagram

A total of 16,911 records were identified through an electronic database search (16,842 records) and other methods (69 records) including the abbreviated search. After removing 5723 duplicate records, 11,188 records were identified for screening. Thirty-two records were excluded as they were published before January 1990. Using title screening, another 10943 records were excluded. The remaining 213 records were screened using abstract and full text with 196 records excluded by the screenings. Abstract screening excluded 124 records with reasons such as (i) participants were not type 2 diabetic (nine studies), (ii) non-experimental study (90 studies), (iii) non-primary care settings, e.g. hospital setting (10 studies), (iv) interventions not related to CCM (14 studies) and (v) non-clinical related outcomes (one study). Full text screening excluded 72 records with reasons such as (i) participants were not type 2 diabetic (seven studies), (ii) non-experimental study (13 studies), (iii) non-primary care settings, e.g. hospital setting (14 studies), (iv) interventions not related to CCM (17 studies), (v) non-clinical related outcomes (three studies) and (vi) limited information on interventions and results (18 studies). Examples of limited information were mainly (i) no information on sample size for groups, (ii) HbA_1c_ levels not available and (iii) means or percentages provided without standard deviations or standard errors for HbA_1c_ levels and other readings. In total, 17 records [53–69] were included for this review (see Fig. 1). The PRISMA flow diagram is illustrated in Additional file 2 and the PRISMA checklist in Additional file 3. The funnel plot appeared symmetrical, suggesting no publication bias (see Additional file 4).

### Study characteristics

The study characteristics of the 17 included studies are summarized in Table 1. These are 17 unique RCTs published between 2008 and 2021 involving 16485 patients. Six studies were from Europe [53, 55, 57, 60, 64, 68], five from the USA [54, 58, 65, 67, 69], four from Asia [56, 61, 62, 66] and one each from Australia [63] and Canada [59]. One study had three groups [67]. The majority of patients were recruited from general practice (GP) or primary care clinics (*n*=14771), while the remaining six were recruited from community health centres (*n*=1714) [54, 61, 63, 65, 67, 69]. The mean age of the participants was 59.3 years, with a range of 45.7 [65] to 71.5 [61]. One trial did not report the age of participants [62]. Seven trials had more males [55–57, 59, 60, 64, 68]. Five interventions were delivered by physicians [56, 57, 59, 64, 68]. Others involved nurses [53, 55, 58, 60], community health workers [54, 63, 65], public health assistants [61], social workers [62], pharmacists and dietitians [66], health educators [67] and behavioural health providers [69]. The majority of interventions lasted 12 months or longer, while five studies lasted less than 12 months [58, 59, 61, 62, 69]. The majority of patients had prevalent diabetes; one trial had newly diagnosed diabetes [64], and another had screen-detected diabetes [60]. Three studies described the CCM elements within the interventions [55, 61, 66]. The CCM interventions within the remaining studies were independently identified and described by two reviewers (LHG and CJRS) based on the description of the CCM elements from the developer [19] (see Table 1). From the 17 studies, there were a total of 64 CCM elements, ranging from two to five elements in each study (see Additional file 5). Eleven studies had four elements [53, 54, 56, 59, 60, 63–66, 68, 69], four studies [57, 58, 62, 67] had two elements and two studies [55, 61] had five elements. One study with two intervention groups contributed two elements each from self-management support and delivery system design [67]. The elements of self-management support and delivery system design were found in the same 16 studies [53–56, 58–69], while decision support was found in 13 studies [53–55, 57, 59–61, 63–66, 68, 69], clinical information systems in 11 studies [53–57, 59–61, 64, 68, 69], organization of healthcare delivery system in four studies [55, 56, 61, 66] and community linkage in two studies [63, 65].

**Table 1:** Characteristics of included studies in the meta-analysis

**Source**	**Setting**	***Total No.***	**Age, years**	**Duration, months, unless stated**	**Outcomes measures and Treatment Targets set**	**Interventions (with CCM elements)**	**Usual care**	**No./Type of CCM elements identified**
Cleveringa et al, [53] 2008 the Netherlands	Primary care practices	3391	I: 65.2 ± 11.3 C: 65.0 ± 11.0	12	HbA_1c_ <7% SBP < 140 mmHg DBP LDL <2.5 mmol/L	Patient diabetes consultation with practice nurse (DSD); Computerized decision support systems (CIS); Diagnostic and treatment algorithm based on national diabetes guidelines (DSD); Patient-specific treatment advice (SMS); Recall system for patients (DSD); Feedback to providers and patients (DS, CIS)	Diabetes care provided by primary care physicians or practice nurse under physician responsibility	4 SMS DS DSD CIS
DePue et al, [54] 2013 American Samoa	Community health centre	268	I: 56 ± 12.5 C: 54 ± 12.9	12	HbA_1c_ SBP DBP BMI	Nurse care manager teaches patient education self-management support and patient-centred communication skills (SMS, DSD); conducts patient groups sessions for high risk patients (DSD); provides feedback to physicians about patient care needs (CIS) Community health workers: ensure patient’s follow-up (DSD), reinforced adherence to medications, problem-solved barriers to self-care, provide support and mobilize family support for diabetes self-support (SMS) Training of staff on standards of care, diabetes guidelines and CCM (DS) Patient care guided by use of protocol or treatment algorithm (DS) Regular reviews by NCM or CHW based on risk profile and patient’s self-selected goals (SMS) Patients get a copy of National diabetes education programme (DS)	Patients on waitlist to join intervention in one year; Received one phone call at 6 months to update contact information, promote study retention and identify adverse effects that occurred since baseline. Patients get a copy of National diabetes education programme	4 SMS DS DSD CIS
Frei et al, [55] 2014 Switzerland	Primary care practices	326	I: 65.7 ± 10.4 C: 68.3 ± 10.6	12	HbA_1c_ ≤ 6.5% SBP <130mmHg DBP <80 mmHg LDL <2.6 mmol/L BMI	Training of practice nurses (OHS) on knowledge of treatment of diabetes patients and general communication skills (DS); empowers nurses to provide structured care for chronically ill patients (DS); perform visits and follow-up consultations using a monitoring tool that guides nurses through consultations with patients and ensures treatment recommendations are followed and used as communication tool with PCPs (DSD) Training of primary care physicians and nurses in 2 workshops on implementation of team approach in practice and evidence-based therapy of diabetes (DS); professional exchanges regarding implementation experience and management of cardiovascular risk factors (CIS) Regular patient consultations with nurse to record parameters for self-management support, makes goals and track progress of treatment recommendations (SMS)	Focussed on PCP and PCP-patient relationship, based on good clinical practice	5 OHS SMS DS DSD CIS
Hayashino et al [56], 2016 Japan	Primary care practices	2236	I: 56.5 ± 5.9 C: 56.5 ± 5.9	12	HbA_1c_ SBP DBP BMI	PCPs: PCPs use a disease management system of monitoring and provided feedback on quality of diabetes care (OHS); PCPs received a monthly report (feedback letter) of their care quality (CIS) Patients: Received reminders for regular visits and lifestyle interventions (DSD); Received patient education (SMS) from diabetes educators, dieticians or nurses on lifestyle changes (information on target body weight, recommended food intake and exercise therapy) by phone or in-person sessions	PCPs provided ordinary medical treatment to their patients	4 OHS SMS DSD CIS
Heselmans et al, [57] 2020 Belgium	Primary care practices	3815	I: 67.2 ± 13.3 C: 64.6 ± 14.7	12	HbA_1c_ SBP DBP LDL	Use of EBMeDs, a computerized decision support system (CIS) that contains evidence-based guidelines designed to improve clinical decision support (DS); and is integrated into the electronic health records Use of Evidence Linker that provides relevant clinical guidelines on demand	Use of Evidence Linker that provides relevant clinical guidelines on demand	2 DS CIS
Hiss et al, [58] 2007 United States	Primary care practices	197	I: 55.7 ± 13.1 C: 57.0 ± 11.4	6	HbA_1c_ SBP DBP	NCM provides: Personal report on basic intervention with explanations; Problem identification with problem-specific, short-term goal setting and development off action plan (SMS); communication with PCP (DSD) regarding initial discussions with patient; advice to patient to contact PCP for follow-up on identified problem.	Received basic intervention (comprehensive baseline evaluation of diabetes with results communicated to patient and PCP)	2 SMS DSD
						Collaborative interaction between nurse, PCP and patient (DSD) leading to short-term goal attainment and experience for patient as active team member (SMS); Proactive and continuous follow-up by NCM		
Holbrook et al, [59] 2009 Canada	Primary care practices	511	I: 61.0 ±13.1 C: 60.5 ±11.9	6	HbA_1c_ <7% SBP <130 mmHg DBP <80 mmHg LDL <2.6 mmol/L BMI <27	PCPs: Web-based diabetes tracker (CIS) with 13 variables based on guidelines (DS); Use tracker to set targets for monitoring process and clinical outcomes Patients: Phone reminders for appointments (DSD); Given access to tracker and mailed hard copy tracker to bring to physician’s consultation and most recent lab results (SMS)	Patients in the control group continued receiving usual care from their respective primary care providers.	4 SMS DS DSD CIS
Janssen et al, [60] 2009 Netherlands	GP practices	498	I: 60.1 ±5.4 C: 59.9 ±5.1	12	HbA_1c_ <7% SBP <120 DBP <80 LDL BMI	GPs trained in treatment protocol (DS) regarding intensive multifactorial treatment for cardiovascular risk using intensified treatment consisted of pharmacological treatment to achieve glucose, blood pressure and lipid targets; combined with structured lifestyle education; GPs are reminded once a year to treat patients according to protocol (CIS) Diabetes nurses trained in management of treatment algorithms and in providing lifestyle education (SMS); authorised to prescribe medications supervised by GPs Patients regularly reviewed seen by GPs and nurses (DSD) Patients referred to internist if targets not reached (DS)	Use of local guidelines No detailed instructions on lifestyle education No further training of GPs after initial symposium No nurse involved in care	4 SMS DS DSD CIS
Kong et al, [61] 2019 China	Community health centres	258	I: 69.1 ±10.5 C: 71.5 ±8.8	9	HbA_1c_ SBP DBP LDL BMI	Health system (OHS): Additional subsidies given to physicians for patient education (enhance patients’ awareness of chronic disease management) and encourage patient initiative through pamphlets and in-person communication) Physicians given appropriate supervision and evaluation procedures Self-management support (SMS): Physicians helped patients in goal-setting, planning, doing, checking and assessing; made self-management plans Decision support (DS): Clinical guidelines implemented by physicians; Physicians received clinical guidelines training and continuing medical education; Physicians received feedback of baseline medical records to better understand care provision Delivery system design (DSD): Each team included a responsible physician, health manager and public health assistant with clear roles and task; Primary role of team is to help patients self-manage their diseases, with monthly follow-up and respond to concerns of patients and other regular tasks	Received conventional follow-up every 3 months by responsible physicians through office visits, home visits and telephone calls Patients examined for lifestyle changes, diabetes control, treatment compliance, drug side effects and target organ damage Patients given general care guidance Physicians received reminders for follow-up every 3 months from tracking system	5 OHS SMS DS DSD CIS
						Clinical information system (CIS): Provided population-based care for patients including tracking, disease management, and assessment; System could share data between community health centres and other healthcare entities such as tertiary care; Patients’ data regularly collected to facilitate care; Physicians received monthly reminders of follow-ups from tracking system; Physicians required to document timely feedback information		
Lee et al, [62] 2011 Hong Kong	General outpatient (primary care) clinics	157	Not reported	28 weeks	HbA_1c_ <6.5% BP BMI	Social worker (DSD) provides self-management programme and assessment (SMS); Programme helps to promote patients’ own problem solving skills; enhanced their self-efficacy on self-management; Used small groups with opportunity for individual advice if needed (DSD)	Attend medical follow-up with general advice on lifestyle and drug compliance.	2 SMS DSD
McDermott et al, [63] 2015 Australia	Community health service	213	I: 47.9 ±10.7 C: 47.8 ±8.9	18	HbA_1c_ SBP DBP LDL	Indigenous health worker resident (DSD) with case management. Indigenous health worker received training in clinical aspects of diabetes including support patients in self-management skills, advice on clinical care, follow-up appointments. Training included (DS) Rationale for Chronic Care Model and evidence-based management and treatment goals in diabetes Hands-on case management	Wait list group. No other description.	4 CL SMS DS DSD
						Working in primary care team with clear roles and responsibilities Engage patients and use local resources to support patient self-management (CL) Evidence-based guidelines and reflective practice (DS) Sharing approaches to problem solving with clinical support team and peers (SMS) Tasks included helping patients understand their medications and nutrition and the effects of smoking and work with the family to help support patient in self-management (SMS)		
Olivarius et al, [64] 2001 Denmark	GP practices	1263	I: 64.9 ±13.9 C: 65.0 ±12.7	6 years	HbA_1c_ SBP DBP	Structured personal care: Regular follow-up and individualized goal setting (SMS) supported by prompting of GPs (DSD), clinical guidelines, feedback and continuing medical education; GPs received descriptive feedback reports on patients (CIS) GPs given seminar on clinical treatment guidelines on diet, smoking, persistent hyperglycaemia, hypertension and hyperlipidaemia (DS) GPs handed out patient leaflets to patients on guidelines (DS)	Routine care by GP in ordinary consultations where GPs are free to choose any treatment and change it over time. No disease management sessions run by nurses.	4 SMS DS DSD CIS
Prezio et al, [65] 2013 United States	Community health services clinic	180	I: 47.9 ±11.0 C: 45.7 ±10.7	12	HbA_1c_ <7% SBP DBP LDL BMI	A culturally tailored diabetes education and case management programme by bilingual community health worker (DSD) along with usual medical care CHW received training in role of diabetes educator and manager Scheduled appointments with patients (DS) Use of printed educational materials targeted for low literacy levels Taught patient education (SMS) including self-monitoring of glucose, meal planning, medication use, sick day rules, smoking cessation, exercise recommendations and information about diabetes complications including recommendations of community resources for exercise (CL) Facilitated physician contact to address acute problems, assisted with pharmacy refills and arranged specialty visits (DS) Physicians follow-up with patients for usual medical care (DSD)	Waitlist group that received usual medical care by physicians Patients provided with glucose monitor and test strips and instructed by medical assistants to use. Patients provided with culturally tailored printed diabetes education materials	4 CL SMS DS DSD
Ramli et al, [66] 2016 Malaysia	Public primary care clinics	888	I: 58 ±0.5 C: 57 ±0.5	12	HbA_1c_ <6.5% SBP <130 mmHg DBP <80 mmHg LDL ≤2.6 mmol/L BMI <23 kg/m^2^	Organisation of Health Care (OHS) and Delivery system design (DSD): Create or strengthened a chronic disease management team (multidisciplinary team led by Family Medicine Specialist to improve coordination of care for type 2 diabetes and co-existing cardiovascular risk factors) Decision support (DS): Use the national Clinical Practice Guidelines for type 2 diabetes to aid management and prescribing; Training provided to intervention team to facilitate and support intervention Self-management support (SMS): Used the Global Cardiovascular Risks Self-Management Booklet to support patients’ self-management	Allied health available but may not be functioning as a team in managing type 2 diabetes. Control clinics have access to Clinical Practice Guidelines but did not receive training and Clinical Practice Guidelines utilisation not emphasized or monitored.	4 OHS SMS DS DSD
Schillinger et al, [67] 2009 United States	Community health network clinics	226 I: 112 C: 114^a^	I: 55.9 ±12.7 C: 55.8 ±11.8	12	HbA_1c_ SBP DBP BMI	ATSM, automated telephone self-management support Patients received automated telephone calls Nurse case management (DSD): Patient responses triggered either immediate, automated health education messages and/or subsequent nurse phone follow-up. Meant to promote self-efficacy, goal-setting and action plans (SMS) All patient interactions and action plans recorded on standardized self-management support records to communicate with patient’s physician	Not described	2 SMS DSD
Schillinger et al, [67] 2009 United States	Community health network clinics	227 I: 113 C: 114	I: 56.5 ±11.4 C: 55.8 ±11.8	12	HbA_1c_ SBP DBP BMI	GMV, group medical visits Uses a group process (DSD) to provide support, education and patient activation (SMS): GMV involved monthly sessions, co-facilitated by a primary care physician and health educator (DSD) Meant to promote self-efficacy, goal-setting and action plans (SMS) All patient interactions and action plans recorded on standardized self-management support records to communicate with patient’s physician (SMS)	Not described	2 SMS DSD
Sonnichsen et al, [68] 2010 Austria	GP practices	1,489	I: 65.4 ±10.4 C: 65.5 ±10.4	12	HbA_1c_ SBP DBP LDL BMI	DMP that consist of: Physician training on diabetes care, current guidelines and practice management training (DS) Patient education in groups (SMS) Standardised documentation of clinical information (physical examination, laboratory findings and diabetes complications) in a DMP form once a year (CIS) Structured interdisciplinary care according to national diabetes guidelines (DS) Agreement on therapeutic goals in a shared patient-physician decision-making process at 3-monthly intervals (SMS)	Physicians performed usual care; Physicians not permitted to participate in DMP training course; Patient education for diabetes publicly available but not explicitly invited to participate Patients put on waitlist	4 SMS DS DSD CIS
Talavera et al, [69] 2021 United States	Community health centre	456	I: 55.4 ±9.8 C: 56.0 ±9.9	6	HbA_1c_ SBP DBP LDL	Co-location of clinical team (physician/mid-level medical provider and specialty behavioural health provider) (DS) Warm hand-off from medical provider to behavioural health provider (DSD) Shared treatment plan (CIS) Up to 4 integrated medical visits with medical provider for medical management of diabetes and other chronic medical conditions and with specialty behavioural health provider for management of psychosocial and behavioural factors (DSD) Care coordination to facilitate shared treatment plan (DSD) Six culturally appropriate, group-health education classes led by community health worker (SMS) All intervention providers are Spanish-English bilingual and Latino/a.	Care follows national consensus guidelines Practitioners stay current through peer review and access to “UpToDate” a point of care clinical support resource; Quarterly primary care visits for patients not on insulin and not meeting treatment goals; Patients are referred to health educator and/or to behavioural health at physician’s discretion; 60% of primary care providers in clinical setting and most of ancillary staff are Spanish-English bilingual and Latino/a.	4 SMS DS DSD CIS

Data are shown as mean ± SD unless stated otherwise

Abbreviations: *BMI* body mass index, *BP* blood pressure, *C* Comparison, *CCM* Chronic Care Model, *CHW* community health worker, *CIS* Clinical Information Systems, *CL* community linkages, *DBP* diastolic blood pressure, *DMP* disease management programmes, *DS* decision support, *DSD* delivery system design, *DBP* diastolic blood pressure, *GP* general practice, *I* intervention, *NCM* nurse care manager, *PCP* primary care physicians, *LDL* LDL cholesterol, *OHS* Organisation of Healthcare Delivery System, *SBP* systolic blood pressure, *SMS* self-management support

^a^ Control group numbers from Schillinger et al was 114 for both arms of the study.

All studies reported the primary outcome (HbA_1c_ level). Nine studies had a mean baseline HbA_1c_ <8% for both groups (range of 6.8 to 7.7%) [53, 55–61, 68], while the remaining eight studies had a mean baseline HbA_1c_ ≥8% for both groups (range of 8.1 to 10.7%) [54, 62–67, 69]. All studies except one [62] reported the secondary outcomes of systolic and diastolic blood pressures. The LDL cholesterol was reported in 11 studies [53, 55, 57, 59–61, 63, 65, 66, 68, 69] and BMI was reported in nine studies [55, 56, 59–61, 65–68].

Usual care of the included studies was broadly described as (i) care provided by primary care physicians or practice nurse following good clinical practice involving routine medical evaluation, patient education on general care, use of home glucose monitoring, patients given a copy of their diabetes test results and follow-up calls to patients after visits [53–55, 58, 61–65, 68, 69] and (ii) physicians have access to relevant clinical guidelines [57, 60, 66]. Three studies described usual care as ordinary medical care by physicians without further descriptions [56, 59, 67].

### Risk of bias

The risk of bias summary and graph are presented in Additional file 6. Eight studies were appraised as unclear risk due to lack of information about random sequence generation [53, 58, 60–64, 67], while 10 studies were graded as unclear risk due to insufficient information about allocation concealment [53, 54, 58, 60–63, 65, 67, 68]. Although it was not feasible to blind participants and personnel due to the nature of the interventions, the absence of blinding did not affect the objective outcomes. Therefore, all 17 studies were graded as low risk for blinding of participants, personnel and outcome assessment [70, 71]. Under incomplete data, three studies [62, 64, 69] were rated as high risk, as ≥20% attrition rate observed in either or both arms posed a serious threat to the study’s validity [72]. One study was rated as unclear risk, as the numbers of participants were not reported at randomization [57]. For selective reporting, 10 studies were assessed as low risk, while seven studies lacked clarity and were hence assessed as unclear risk [53, 54, 58, 59, 61, 64, 69].

### Effectiveness of CCM

#### HbA_1c_

**Fig. 2:**
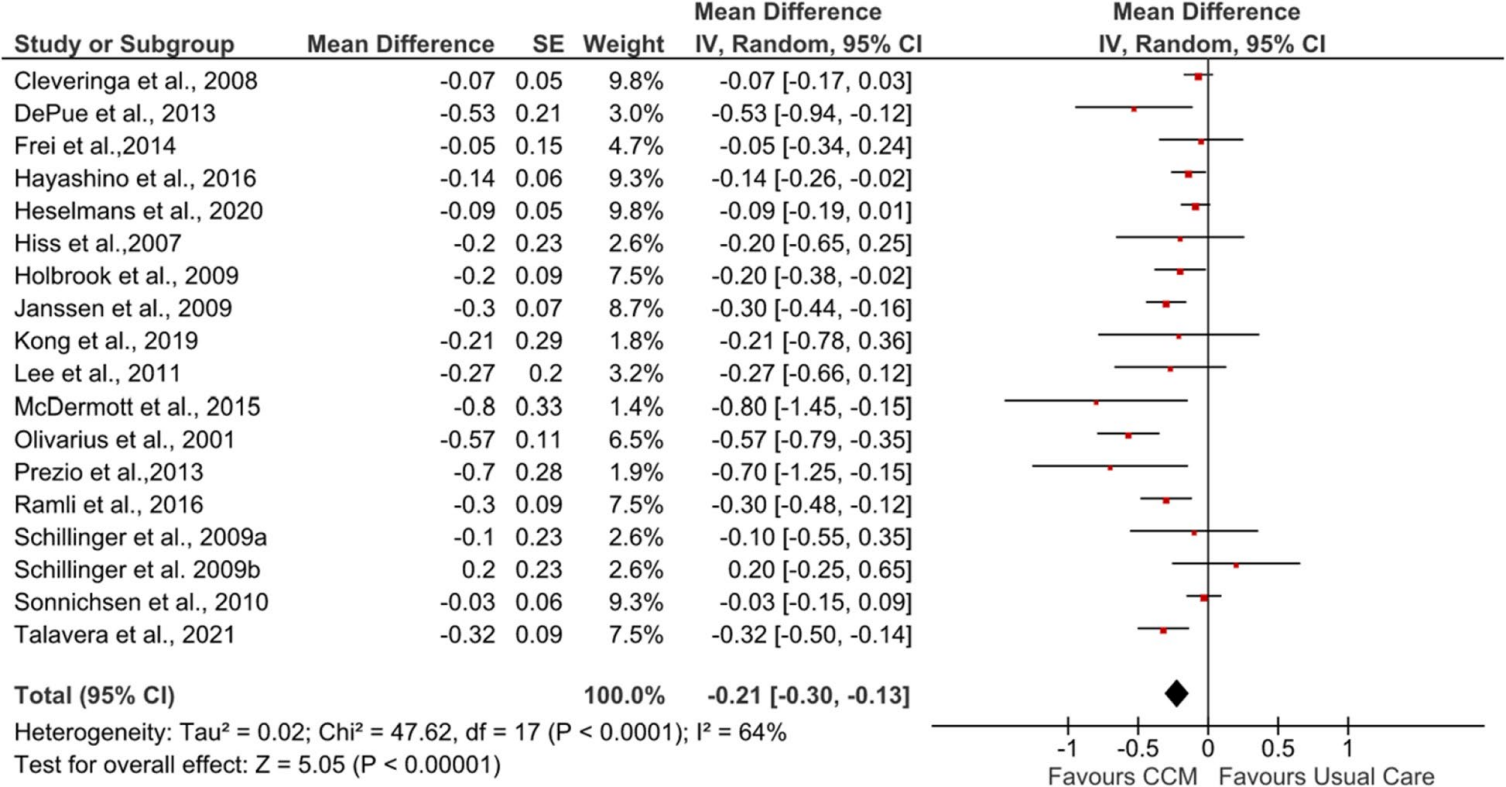
Forest plot showing the effect of CCM on post-intervention HbA_1c_ (%). IV, inverse variance

All 17 studies that assessed the effect of CCM (intervention) vs usual care (control group) on postintervention HbA_1c_ levels were pooled into the meta-analysis (see Fig. 2). Compared with usual care, adults who received CCM interventions had significantly improved HbA_1c_ levels (MD −0.21%, 95% CI −0.30, −0.13; *Z* = 5.07, *p*<0.00001).

#### Blood pressure

**Fig. 3:**
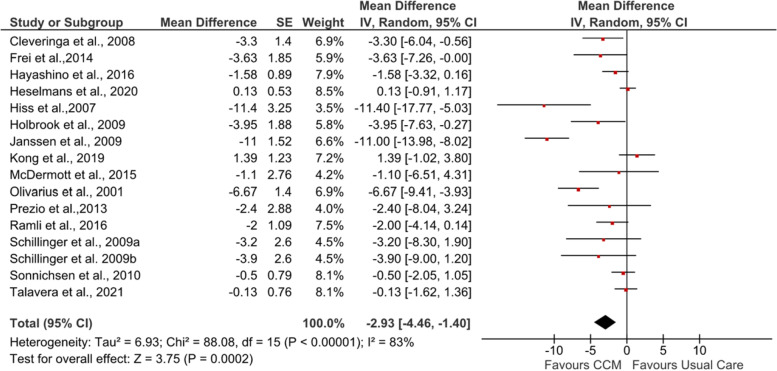
Forest plot showing the effect of CCM on post-intervention SBP (mmHg). IV, inverse variance

**Fig. 4:**
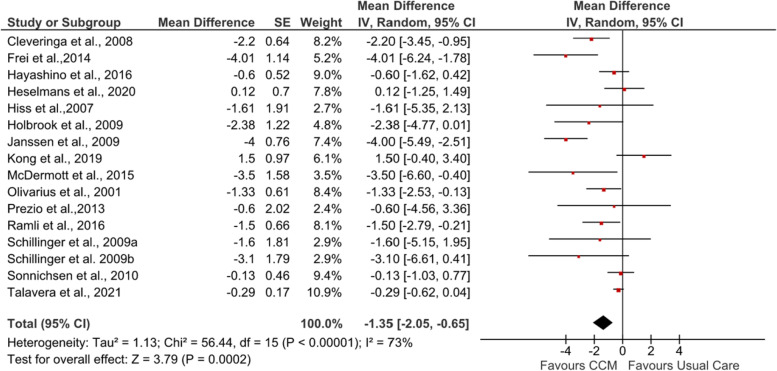
Forest plot showing the effect of CCM on post-intervention DBP (mmHg). IV, inverse variance

The pooled results of 15 studies [53, 55–61, 63–69] for SBP showed a significant improvement (MD −2.93 mmHg [95% CI −4.46, −1.40]; *Z* = 3.75, *p*=0.0002) (see Fig. 3). Two studies [58, 60] showed a large improvement in SBP (MD −11.4 and −11 mmHg, respectively) compared to other studies. For DBP, the pooled results also presented a statistically significant improvement (MD −1.35 mmHg [95% CI −2.05, −0.65]; *Z* = 3.79, *p*=0.0002) (see Fig. 4). The study with newly diagnosed patients [64] showed improvement in both SBP (MD −6.67 mmHg [95% CI −9.41, −3.93]) and DBP (MD −1.33 mmHg [95% CI −2.53, −0.13]) after six years of intervention.

#### LDL cholesterol levels

Pooled results from 11 trials among [53, 55, 57, 59–61, 63, 65, 66, 68, 69] reported an improvement between groups (MD −0.07 mmol/L [95% CI −0.16, 0.02]; *Z* = 1.62, *p*=0.11) (see Additional file 7). The study with screen-detected patients [60] reported an improvement of MD −0.5 mmol/L [95% CI −0.66, −0.34] in favour of the intervention group.

#### BMI

Pooled data from nine studies [55, 56, 59–61, 65–68] showed an improvement in BMI between groups (MD −0.14 kg/m^2^ [95% CI −0.29, 0.01]; *Z* = 1.78, *p*=0.08) (see Additional file 8). The Austrian study [68] had the largest reduction in change in BMI between the groups (MD −0.53 kg/m^2^ [95% CI −1.04, −0.02]).

### Subgroup analysis

Subgroup analyses were performed for the primary outcome HbA_1c_ level. Subgroup analyses were stratified by participants’ mean baseline HbA_1c_ levels, study duration and number of CCM elements in the interventions. Subgroup analysis revealed no significant subgroup difference for study duration (<12 months vs ≥12 months) (*p*=0.55) (see Additional file 9).

**Fig. 5:**
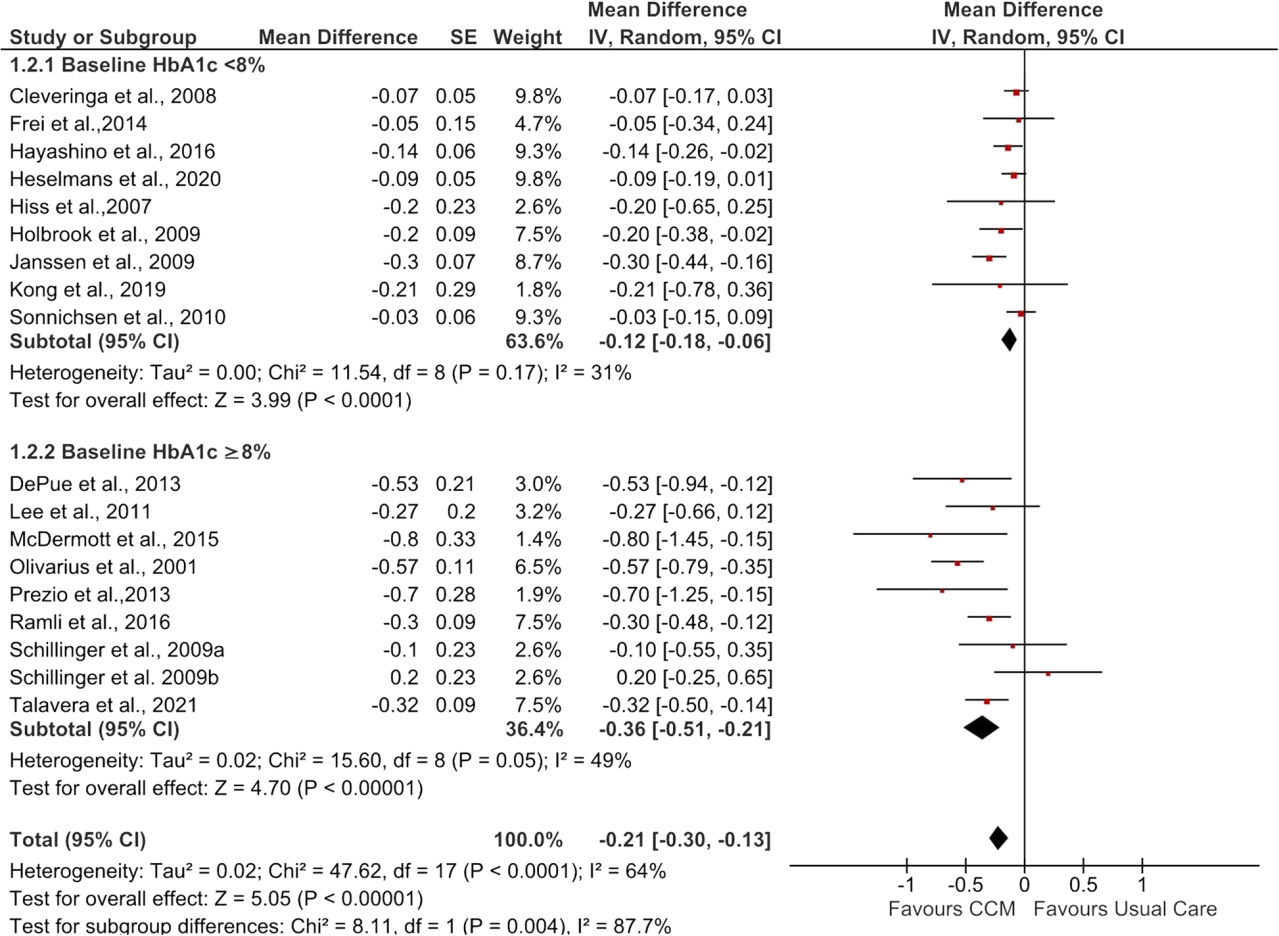
Forest plot showing subgroup analysis of post-intervention HbA_1c_ (%) according to baseline HbA_1c_. IV, inverse variance

Subgroup analysis comparing the effectiveness of CCM among participants with a mean baseline HbA_1c_<8% and a mean baseline HbA_1c_ ≥8% reported a significant subgroup difference (*I*^2^ = 87.7%, *p*=0.004) (see Fig. 5). Among participants with a mean baseline HbA_1c_ ≥8%, those who received CCM interventions experienced significant reductions in HbA_1c_ levels (MD −0.36%, 95% CI −0.51, −0.21; *Z* = 5.05, *p*<0.00001) compared with participants who received usual care. Similarly, CCM interventions significantly decreased HbA_1c_ levels in participants with a mean baseline HbA_1c_ <8% (MD −0.12%, 95% CI −0.18, −0.06; *Z* = 3.99, *p*<0.0001).

**Fig. 6:**
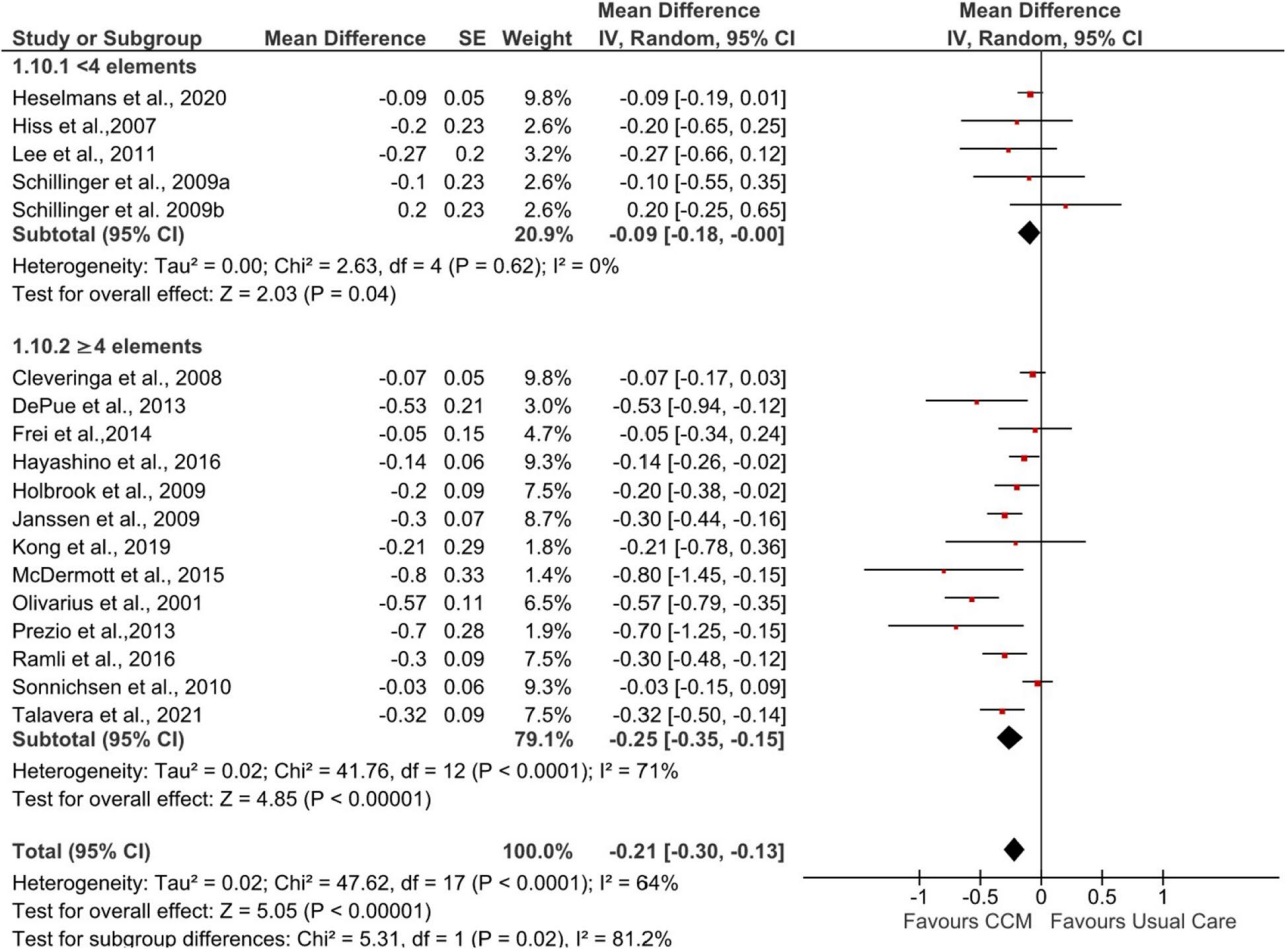
Forest plot showing subgroup analysis of post-intervention HbA_1c_ (%) according to numbers of CCM elements. IV, inverse variance

Additionally, subgroup analysis comparing the effect of CCM among participants who received fewer than four CCM elements and four or more CCM elements in the interventions revealed significant subgroup differences (*I*^2^ = 81.2%, *p*=0.02) (see Fig. 6). Compared with usual care, a significant reduction in HbA_1c_ levels was experienced by participants receiving interventions containing four or more CCM elements (MD −0.25%, 95% CI −0.35, −0.15; *Z* = 4.85, *p*<0.00001) and fewer than four CCM elements (MD −0.09%, 95% CI −0.18, −0.00; *Z* = 2.03, *p*=0.04).

## Discussion

This review assessed the effectiveness of the Chronic Care Model (CCM) for adults with type 2 diabetes in primary care on improving patient outcomes. Our results revealed that, in comparison to usual care, CCM interventions in primary care significantly improved HbA_1c_ and systolic and diastolic blood pressures but not LDL cholesterol or BMI.

The CCM is an integrated model that has been shown to be an effective framework for improving the quality of diabetes care through the implementation of its six core elements [73]. A five-year prospective cohort study using a multidisciplinary Risk Assessment and Management Programme-Diabetes Mellitus (RAMP-DM) for diabetic patients [74] incorporating CCM elements of risk-stratified care planning, multidisciplinary care, scheduled monitoring of complications, diabetes self-management education and smoking cessation showed significant improvements for HbA_1c_, systolic blood pressure, diastolic blood pressure, LDL cholesterol and body mass index readings between groups. Patients enrolled in the CCM intervention experienced a reduction in cardiovascular risks by 56.6%, microvascular complications by 11.9% and mortality by 66.1%. A 12-month randomized controlled trial involving a comprehensive diabetes programme that incorporated risk stratification, action planning, regular follow-up and patient engagement in self-care, improved HbA_1c_ and blood pressure levels for adults with diabetes in primary care clinics within a managed care organization [75]. The RAMP-DM programme also found that the CCM was a cost-saving intervention in managing diabetes in patients over five years [9].

### HbA_1c_ outcomes

Previous systematic reviews that included a meta-analysis [24, 25, 28–30, 36] similarly reported HbA_1c_ reduction between intervention and usual care groups ranging from MD −0.07% (95% CI −0.10, −0.04) [29] to MD −0.5% (95% CI −0.6, −0.3) [28], thus supporting our findings. Our review included nine new studies [57–62, 66, 67, 69] from six search databases and four continents, when compared with another review [30] that shared eight common studies [53–56, 63–65, 68]. However, our current findings revealed a smaller effect estimate of HbA_1c_ decrease of MD −0.21%, 95% CI −0.30, −0.13; *Z* = 5.07, *p*<0.00001, compared with the prior review that reported a HbA_1c_ decrease of MD −0.28% (95% CI −0.35, −0.21) (*p* value not available) [30].

The HbA_1c_ remains a recommended and relevant measure for the medical evaluation and clinical management of people with type 2 diabetes with the goals of treatment being to prevent or delay complications [20]. The UK Prospective Diabetes Study (UKPDS) [22] emphasized the importance of improving glycaemic control in order to reduce diabetes related microvascular complications in people with type 2 diabetes. A 2019 cohort study of 34,737 newly diagnosed patients [21] found that longer periods of early glycaemic exposure at HbA_1c_ levels 6.5 to <8.0% did not increase the risk of microvascular or macrovascular events during follow-up (the Legacy Effect), whereas longer periods of exposure to HbA_1c_ levels ≥8.0% were associated with an increasing risk of microvascular events. Moreover, HbA_1c_ levels ≥9.0% for early exposure periods >0–4 years were associated with an increasing risk of macrovascular events. Similarly, a 10-year observational follow-up of people with type 2 diabetes [76] who were originally randomized to intensive glycaemic control had significant long-term reductions in myocardial infarction (15 to 33%) and in all-cause mortality (13 to 27%) depending on whether sulfonylurea or insulin or metformin was given as initial pharmacotherapy.

The Action to Control Cardiovascular Risk in Diabetes (ACCORD) study found that the use of intensive therapy to target HbA_1c_ below 6% for 3.5 years in patients with type 2 diabetes who have established cardiovascular disease or additional cardiovascular risk factors, increased mortality and did not significantly reduce major cardiovascular events [77]. Although the 2022 ADA guidelines [78] recommends that the HbA_1c_ goal of <7% is appropriate for many non-pregnant adults without significant hypoglycaemia, there are different recommendations for adults with limited life expectancy or who are older [48]. Older adults who are otherwise healthy with few coexisting chronic conditions, intact cognitive function or function status are recommended to work towards lower glycaemic goals such as HbA_1c_ < 7.0–7.5%. On the other hand, it could be more appropriate for older adults who have multiple coexisting chronic conditions, cognitive impairment or functional dependence or people with limited life expectancy to aim for less stringent glycaemic goals such as HbA_1c_ < 8.0%.

In setting the glycaemic targets, the studies in this review used guideline recommendations and thus demonstrating the presence of the decision support element from the CCM. Two studies in this review [58, 64] were conducted before the ACCORD study results were published and were based on prevailing international guidelines. Seven studies measured specific HbA_1c_ goals [53, 55, 59, 60, 62, 65, 66] with majority targeting 7% or below and three studies targeting 6.5% or below [55, 62, 66]. Five of these seven studies based their recommended targets on national guidelines [53, 66] or international guidelines such as the ADA guidelines [55, 65] or both [59], while the remaining two studies did not mention what was the source for the recommended targets [60, 62]. In total, nine studies in this review did not specify any target HbA_1c_ goals as outcomes [54, 56–58, 61, 63, 67–69].

Though guideline recommendations for health care providers are tools that can be used to improve health outcomes, diabetes care should be individualized for each person in order to achieve optimal outcomes. Taking a CCM patient-centred care approach, glycaemic goals are recommended to be personalised based on the individual’s medical conditions and preferences [79]. The element of delivery system design from the CCM advocates for collaborative, multidisciplinary teams to provide care for people with chronic diseases such as diabetes and to facilitate patients’ self-management. The self-management system element from the CCM addresses the need for goal-setting and helps clinicians incorporate personalised HbA_1c_ goal-setting in the management of patients with type 2 diabetes.

In this review, two studies demonstrated the self-management system element by having personalised or realistic goals for glycaemic control for different patients [54, 64] while the remaining studies were less clear if personalised goals were set. In the first study, the intervention protocol was guided by a treatment algorithm (an aspect of the decision support element in CCM) that determined the frequency and intensity of patient care, based on level of diabetes control and patient’s associated health risks including the use of higher cut-points for HbA_1c_ goals [54]. This study also demonstrated how the elements of decision support and self-management support were intertwined in the interventions using the CCM. In the second study, the intervention group incorporated realistic and best possible goals for glycated haemoglobin, blood pressure, and lipids within predefined categories [64]. In the remaining studies, three described aspects of patient-centred care such as patient-specific goal setting for glycaemic status, blood pressure and serum lipids [55], agreement on therapeutic goals in a shared patient-physician decision-making process [68] and shared treatment plan, shared decision-making and goal-setting [69]. Our review shows that the CCM can be used to address the gap in the lack of personalised goal-setting for the management of patients with type 2 diabetes, in particular for those who may experience adverse outcomes with intensive blood glucose control [77].

In our review, all except one trial contained the element of self-management support (see Table 1 and Additional file 5). This trial looked at computerized clinical decision support for providers and did not contain the element of self-management support for patients [57]. A systematic review by Si et al [25] reported that RCTs involving the self-management support element had a reduction in HbA_1c_ by −0.53% compared to usual care. The self-management support element of the CCM emphasizes that the patient is the main person responsible for managing their health and the healthcare provider works with the patient to jointly identify problems, set goals, establish priorities, and develop an action plan and strategy for solving the problems that have been identified. Empowering patient self-management is fundamental to the successful implementation of the CCM [18]. It is therefore not surprising that almost all studies in our review incorporated self-management support in the interventions. Other commonly occurring elements found in this review were decision support, delivery system design and clinical information systems, while the elements of organizational system design and community linkages were least observed (see Additional file 5), as similarly reported in other reviews [24, 25, 28, 33, 34, 80, 81]. Nonetheless, our review was not able to show that any single CCM element was found to be critical [24] or superfluous [82] to improve outcomes.

The CCM is about person-centred approach to managing chronic conditions such as diabetes. Therefore, HbA_1c_ should not be the only relevant measure in the management of people with type 2 diabetes nor be the key determinant of whether health systems should adopt the CCM or not. There should be focus on non-biochemical outcomes such as quality of life and reduction of complications, which have value in themselves, even if the HbA_1c_ outcome did not come down. In this review, five studies measured quality of life as an outcome [55, 59–61, 67] with three studies [55, 60, 61] using the 36-item short-form health survey (SF-36) [83], and two studies [59, 67] using SF-12 [84]. An additional measure, Diabetes-39 questionnaire was used to assess diabetes-related quality of life [85, 86] for one study [59]. Four studies [55, 59, 60, 67] showed there was no statistically significant change in quality of life measures between groups though there was a positive trend in one study [59]. One study [61] showed statistically significant increases in four scales of the SF-36, namely, the role limitation due to physical problems and social functioning, the role limitation due to emotional problems and the physical component summary score.

### Blood pressures and BMI outcomes

Looking at the effect of CCM interventions on the secondary outcomes in this review, adults with type 2 diabetes and hypertension were found to have improved systolic and diastolic blood pressures. A probable reason could be the patients’ ability to perform self-management. Those who are able to perform their glucose or blood pressure measurement readily at home to enable self-monitoring would gain better control of their condition [87, 88]. Conversely, patients with hyperlipidaemia were only able to assess their progress through blood tests in the clinics and hence it may reduce their level of self-management. More research is recommended to affirm this finding. Other reviews also found small gains in cholesterol improvement that could be clinically trivial [29, 30, 36]. Our study also did not find any improvement in BMI.

### Subgroup analyses

#### Baseline HbA_1c_ levels

The baseline HbA_1c_ level was shown in this meta-analysis to affect HbA_1c_ outcomes, with the group having a mean baseline HbA_1c_ ≥8% showing greater reductions in HbA_1c_ changes than the <8% group. This finding concurs with three other meta-analyses [26, 27, 37] except one [30]. A 2017 systematic review [89] established that the HbA_1c_ measurement is a reliable risk factor of all-cause and cardiovascular mortality in diabetics and non-diabetics. The review recommended that the optimal HbA1c levels for the lowest all-cause and cardiovascular mortality were 6.0 to 8.0% in people with diabetes and 5.0 to 6.0% in those without diabetes. In addition, a 2019 cohort study demonstrated that longer periods of exposure to HbA_1c_ levels ≥8.0% were associated with increasing microvascular and mortality risk [21]. Another study targeting the patients with a higher HbA_1c_ level showed that there were benefits from having fewer major cardiovascular events [90]. Our results suggest that CCM interventions could be strategically targeted on patients with HbA_1c_ levels ≥8.0% instead of those with lower readings.

#### Numbers of CCM elements

The majority of the studies in this review contain four or more CCM elements. A greater number of CCM elements was found to have better improvements in HbA_1c_ levels in this review, which is consistent with previous reviews suggesting a greater benefit of interventions with more CCM elements over a single element for type 2 diabetes [28, 29, 91–93], while other reviews did not show the benefits or were inconclusive [24, 25, 73, 81, 82]. Elissen et al. [28] found that the most notable improvement in HbA_1c_ of −0.7% (95% CI −1.2, −0.3, *p*=0.22) was attained by trials having at least three CCM elements. Conversely, having two and fewer CCM elements may reduce the opportunities for education sessions, assessment of the patients’ needs and identifying barriers to self-management [28]. While CCM has been promoted as a package of interventions supported by evidence that interventions with multiple elements do better than single ones, it is challenging to standardize the combinations of CCM elements [28]. More research is needed to provide evidence for supporting synergistic effects than the sum of the parts, with the CCM elements being interdependent and building on one another [14, 82].

#### Length of intervention

Our review did not find any difference in mean HbA_1c_ reduction between trials lasting <12 months vs ≥12 months, similar to other meta-analyses [27, 28, 37]. This could be due to the few studies in the <12 months group in our review, resulting in an uneven distribution of the covariates [94]. Moreover, two studies in this review were much longer than 12 months with one lasting 18 months [63] and the other lasting six years [64]. One study was shorter than six months at 28 weeks [62]. These studies were included in the meta-analysis because there is no recommended duration for CCM to be carried out. Pimouguet et al. [27] and Elissen et al [28] found that studies shorter than 12 months were found to report more promising effects on glycaemic control than those longer than 12 months, although the difference did not achieve statistical significance, while Murphy et al. [37] found no difference. Other meta-analyses using groups of ≤12 months vs >12 months also did not find any significant difference in mean HbA_1c_ reduction between groups [30, 36], though the reduction was greater in the >12 months duration group. Pimouguet et al [27] reasoned that effective features of disease management (ability of disease managers to start or modify medical treatment) could have impacted outcomes, irrespective of study duration or baseline HbA_1c_ levels.

Our meta-analysis shows that studies <12 months [58, 59, 61, 62, 69] showed a significant improvement results while those 12 months [53–57, 60, 65–68] and longer [63, 64] may suggest sustainability of HbA_1c_ improvement using CCM. Diabetes is a long-term condition and it is important to show that CCM interventions can sustain the improved glycaemic control over the longer duration of its management.

This review has limitations. First of all, the majority of studies did not classify the CCM elements in the interventions. Although the two reviewers independently determined the numbers and type of CCM elements in the studies using a guide from the CCM developers, this could still lead to misclassification bias [95]. The varying numbers and types of CCM elements added to the heterogeneity, which could also be explained by the different intervention durations and baseline HbA_1c_ levels in the studies. Information about the randomization procedure and allocation concealment from healthcare providers was often missing, thus affecting the methodological quality of this review. There is a broad variety of usual care for the studies being reviewed and it may not be possible to have a standard of care that is similar for all studies. The review also focussed on improvements in biomedical parameters, not other outcomes such as quality of life. However, measuring biomedical outcomes remains important from some perspectives, as they are predictors of diabetes-related complications which in turn, are key determinants of healthcare costs and quality of life downstream. In this review, only two studies incorporated personalised goals as part of their intervention for diabetes management [54, 64]. Most of the studies identified used a fixed goal for HbA_1c_ rather than personalised goals as would be recommended today, based on evidence of adverse effects of intensive control in some individuals with type 2 diabetes [77]. Therefore, more research is needed to evaluate the effectiveness of personalised goals in diabetes management. While our analysis shows that application of the CCM can result in changes in biomedical parameters in the intended direction, there is no direct empirical evidence that CCM would achieve the same impact in the context of personalized goals. Lastly, the effects of the interventions on biomedical parameters are relatively small and in themselves may not be clinically significant as compared to other interventions like pharmacological therapy which typically lower HbA_1c_ levels 0.5–1%.

More research on CCM interventions is needed for adults with hyperlipidaemia and those who are overweight or obese, as these are not found to be significant in this study. Future research investigating the effectiveness of CCM should clearly classified the elements in the interventions and the descriptions of each CCM element should be better standardized. Research should also measure the effectiveness of the different CCM elements by themselves. Other CCM elements that are not well explored such as the the organization of the healthcare delivery system and community linkages should be investigated in future research. It is recommended for future studies that usual care be clearly described as being different from the interventions. The duration of the CCM intervention should also be investigated for its effectiveness on the outcomes, preferably for longer than 12 months to see if the effects can be sustained. Lastly, while it remains important to measure biochemical outcomes such as HbA_1c_, in particular by setting personalized targets, other measures looking at patient-centred care such as quality of life, reduction of complications and quality of care should also be examined.

## Conclusions

This systematic review and meta-analysis suggests that CCM is effective in primary care adults with type 2 diabetes for HbA_1c_ and blood pressure outcomes. However, CCM interventions did not significantly affect LDL cholesterol or BMI. While there was a greater reduction in HbA_1c_ levels when four or more CCM elements were used in the interventions compared with fewer elements, there was no influence of study duration on HbA_1c_ levels. The elements of self-management support, decision support, delivery system design and clinical information systems were found to be most commonly used in the interventions.

## Supplementary Information

**Additional file 1. **PubMed Search Strategy.

**Additional file 2. **PRISMA 2020 flow diagram.

**Additional file 3. **PRISMA 2020 Checklist.

**Additional file 4. **Funnel plot on HbA_1c_.

**Additional file 5. **Table with numbers of CCM elements found in the interventions for each trial.

**Additional file 6. **Risk of bias.

**Additional file 7. **Forest plot showing the effect of CCM on post-intervention LDL cholesterol (mmol/L). IV, inverse variance.

**Additional file 8. **Forest plot showing the effect of CCM on post-intervention BMI (kg/m^2^). IV, inverse variance.

**Additional file 9. **Forest plot showing subgroup analysis of post-intervention HbA_1c_ (%) according to study duration. IV, inverse variance.

## Data Availability

All data generated or analysed during this study are included in this published article and its supplementary information files.

## Abbreviations

ACCORD: Action to Control Cardiovascular Risk in Diabetes
ADA: American Diabetes Association
BMI: Body mass index
CCM: Chronic Care Model
CI: Confidence interval
DBP: Diastolic blood pressure
GP: General practice
HbA_1c_: Glycated haemoglobin
IV: Inverse variance
LDL: Low-density lipoprotein
MD: Mean difference
MESH: Medical Subject Headings
PRISMA: Preferred Reporting Items for Systematic reviews and Meta-Analyses
PROSPERO: International Prospective Register of Systematic Reviews
RAMP-DM: Risk Assessment and Management Programme-Diabetes Mellitus
RCT: Randomized controlled trial
RevMan: Review Manager
SBP: Systolic blood pressure
SD: Standard deviation
SF-12: 12-item short-form health survey
SF-36: 36-item short-form health survey
UKPDS: UK Prospective Diabetes Study
USD: US dollar
