# Supplementary material for: Effectiveness of the chronic care model for adults with type 2 diabetes in primary care: a systematic review and meta-analysis
Source: Syst Rev. 2022 Dec 15;11:273. doi: 10.1186/s13643-022-02117-w (PMC9753411; doi:10.1186/s13643-022-02117-w)
Supplement: Supplementary file 1 — Additional file 1. PubMed Search Strategy. [file 13643_2022_2117_MOESM1_ESM.docx]

Additional files

Additional file 1: PubMed Search Strategy

| Database |  | | Index and Keywords |  |
| --- | --- | --- | --- | --- |
| PubMed | #1 | "Diabetes Mellitus, Type 2"[Mesh] OR ((type 2[Title/Abstract] OR type II[Title/Abstract] OR type2[Title/Abstract] OR typeII[Title/Abstract] OR non insulin depend*[Title/Abstract] OR noninsulin depend*[Title/Abstract] OR maturity onset[Title/Abstract] OR maturity-onset[Title/Abstract] OR slow-onset[Title/Abstract] OR adult onset[Title/Abstract] OR stable[Title/Abstract]) AND (diabet*[Title/Abstract])) OR MODY[Title/Abstract] OR NIDDM[Title/Abstract] OR T2DM[Title/Abstract] OR T2D[Title/Abstract] | | |
|  | #2 | "Models, Theoretical"[Mesh] OR model*[Title/Abstract] OR theor*[Title/Abstract] | | |
|  | #3 | "Disease Management"[Mesh] OR "Patient Care Team"[Mesh] OR "Patient-Centered Care"[Mesh] OR "Patient Care Management"[Mesh] OR "Self Care"[Mesh] OR "Self Efficacy" [Mesh] OR "Delivery of Health Care"[Mesh] OR "Self-Management"[Mesh] OR "Chronic Disease"[Mesh] OR chronic care [Title/Abstract] OR chronic disease*[Title/Abstract] OR chronic illness*[Title/Abstract] | | |
|  | #4 | #1 AND #2 AND #3 | | |
