# Supplementary material for: Effectiveness of the chronic care model for adults with type 2 diabetes in primary care: a systematic review and meta-analysis
Source: Syst Rev. 2022 Dec 15;11:273. doi: 10.1186/s13643-022-02117-w (PMC9753411; doi:10.1186/s13643-022-02117-w)
Supplement: Supplementary file 2 — Additional file 2. PRISMA 2020 flow diagram. [file 13643_2022_2117_MOESM2_ESM.docx]

**Identification of studies via other methods**

**Identification of studies via databases and registers**

Records identified through database search:

PubMed: 5225, Embase: 5957, CINAHL: 1744, Cochrane: 14, Scopus: 2484, Web of Science: 1418

Total: N = 16842

*All records published to 11/7/2021 were screened

Records identified to 17/11/2021:

- Clinical Trials database search (n = 4)
- Citation searching (n= 60)
  - Three SR (n=25)
  - Twelve RCT papers (n=35)

Records of abbreviated search from PubMed, Embase and CINAHL from 1/1/2021 to 28/4/2022 (n = 5)

Total: N = 69

Records removed before screening (n = 5700)

- Duplicate records removed (n = 3566)
- Records marked as duplicates by automation tools (n = 2134)
- Records removed for other reasons (n = 0)

Duplicate records removed

(n = 23)

- Citation searching (n = 22)
- Clinical trials registry (n = 1)

**Identification**

Records excluded (n = 3) based on title screening

- Clinical Trials Registry n = 3
  - - Non-CCM related based on titles (n = 3)

Records excluded:

- Records before 01/01/1990 (n = 32)
- Irrelevant based on title (n = 10940)

Records screened

(n = 46)

Records screened

(n = 11142)

Records not retrieved based on full text (n = 38)

- - Not type 2 diabetic (n = 6)
  - Non-experimental study (n = 7)
  - Non-primary care setting (n = 10)
  - Non-CCM related (n = 4)
  - Limited information on intervention and results (n = 11)

Records sought for retrieval

(n = 43)

Records not retrieved based on abstract (n = 124)

- - Not type 2 diabetic (9)
  - Non-experimental study (90)
  - Non-primary care setting (10)
  - Non-CCM related (14)
  - Non-clinical related outcomes (1)

Records sought for retrieval

(n = 170)

**Screening**

Records not retrieved based on full text (n = 34)

- - Not type 2 diabetic (n = 1)
  - Non-experimental study (n = 6)
  - Non-primary care setting (n = 4)
  - Non-CCM related (n = 13)
  - Non-clinical related outcomes (n = 3)
  - Limited information on intervention and results (n = 7)

Records assessed for eligibility

(n = 46)

Records included from databases (n = 12)

Records included studies from other methods (n = 5)

**Included**

*From:*  Page MJ, McKenzie JE, Bossuyt PM, Boutron I, Hoffmann TC, Mulrow CD, et al. The PRISMA 2020 statement: an updated guideline for reporting systematic reviews. BMJ 2021;372:n71. doi: 10.1136/bmj.n71. For more information, visit: <http://www.prisma-statement.org/>
