# Supplementary figures and images for: Effectiveness of the chronic care model for adults with type 2 diabetes in primary care: a systematic review and meta-analysis
Source: Syst Rev. 2022 Dec 15;11:273. doi: 10.1186/s13643-022-02117-w (PMC9753411; doi:10.1186/s13643-022-02117-w)

Additional file 4: Funnel plot on HbA1c

**
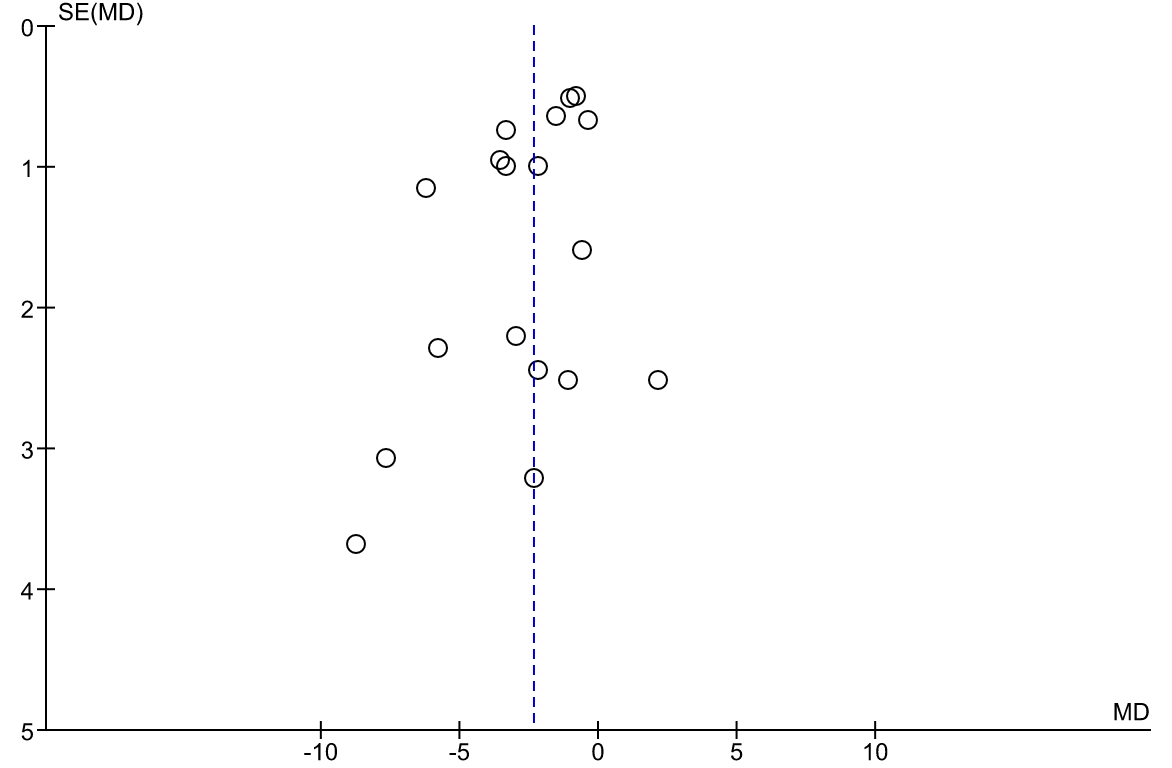
**

Supplement: Supplementary file 4 — Additional file 4. Funnel plot on HbA1c. [file 13643_2022_2117_MOESM4_ESM.docx]

Additional file 6: Risk of bias

**
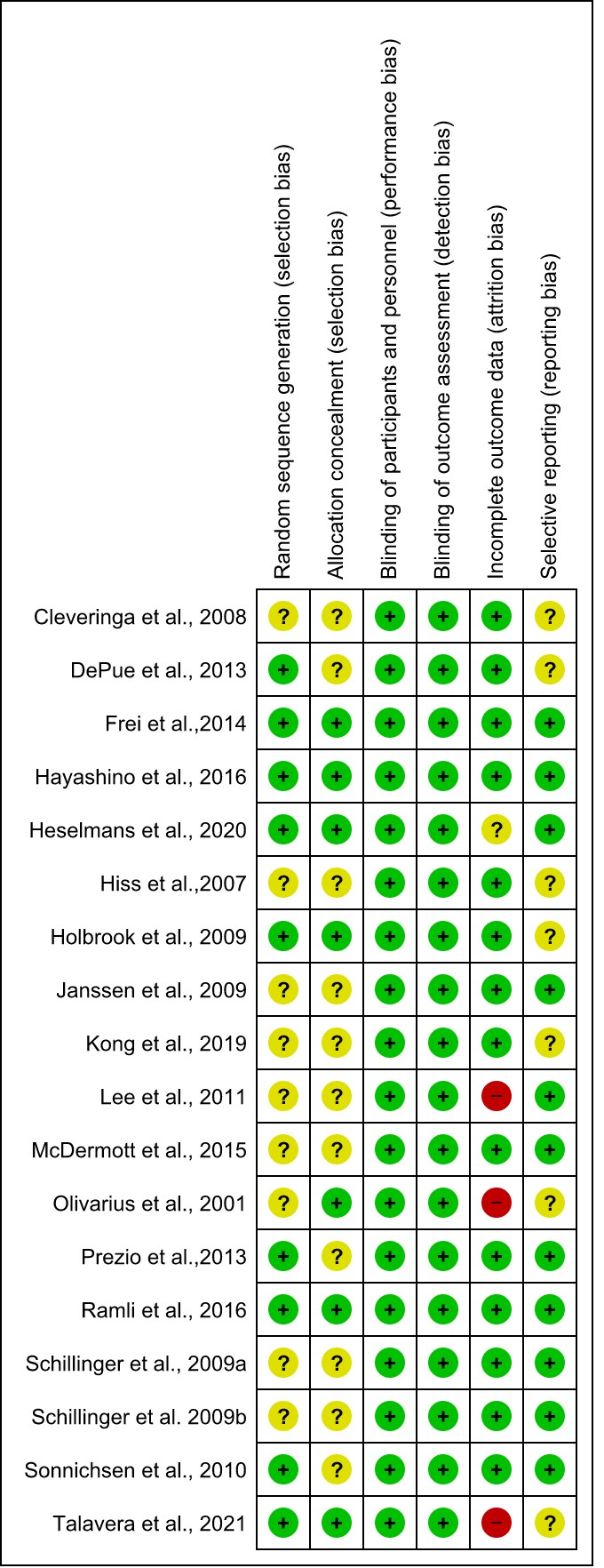
**

(i) Risk of bias summary


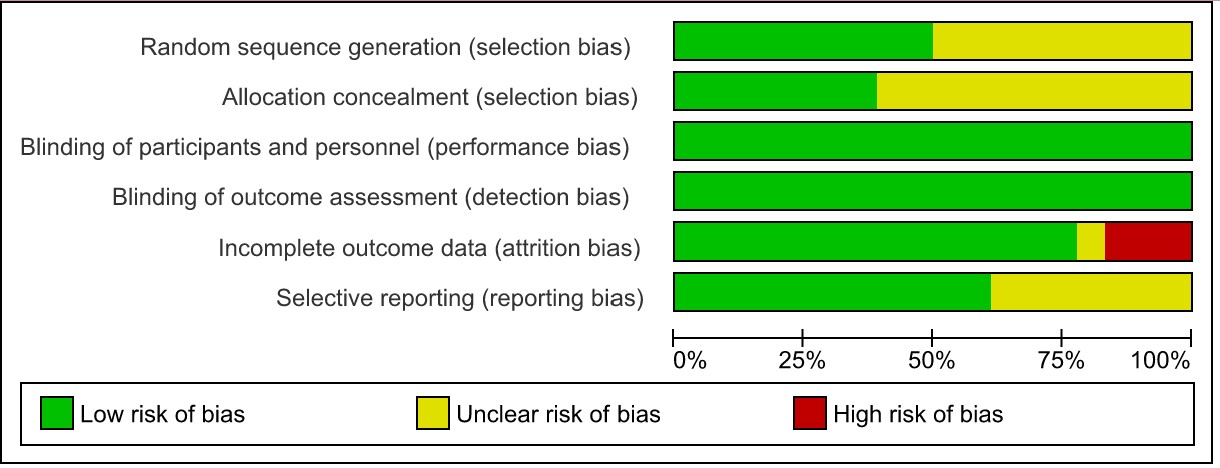


(ii) Risk of bias graph

Supplement: Supplementary file 6 — Additional file 6. Risk of bias. [file 13643_2022_2117_MOESM6_ESM.docx]
