# Supplementary material for: Effectiveness of the chronic care model for adults with type 2 diabetes in primary care: a systematic review and meta-analysis
Source: Syst Rev. 2022 Dec 15;11:273. doi: 10.1186/s13643-022-02117-w (PMC9753411; doi:10.1186/s13643-022-02117-w)
Supplement: Supplementary file 5 — Additional file 5. Table with numbers of CCM elements found in the interventions for each trial. [file 13643_2022_2117_MOESM5_ESM.docx]

Additional file 5: Table with numbers of CCM elements found in the interventions for each trial

| **Records** | **Organization of Healthcare Delivery System** | **Community**  **Linkage** | **Self-management support** | **Decision**  **Support** | **Delivery System**  **Design** | **Clinical Information**  **System** | **Number of Elements** |
| --- | --- | --- | --- | --- | --- | --- | --- |
| Cleveringa 2008 |  |  | ✓ | ✓ | ✓ | ✓ | 4 |
| DePue  2013 |  |  | ✓ | ✓ | ✓ | ✓ | 4 |
| Frei  2014 | ✓ |  | ✓ | ✓ | ✓ | ✓ | 5 |
| Hayashino  2016 | ✓ |  | ✓ |  | ✓ | ✓ | 4 |
| Heselmans 2020 |  |  |  | ✓ |  | ✓ | 2 |
| Hiss  2007 |  |  | ✓ |  | ✓ |  | 2 |
| Holbrook 2009 |  |  | ✓ | ✓ | ✓ | ✓ | 4 |
| Janssen  2009 |  |  | ✓ | ✓ | ✓ | ✓ | 4 |
| Kong  2019 | ✓ |  | ✓ | ✓ | ✓ | ✓ | 5 |
| Lee  2011 |  |  | ✓ |  | ✓ |  | 2 |
| McDermott 2015 |  | ✓ | ✓ | ✓ | ✓ |  | 4 |
| Olivarius 2001 |  |  | ✓ | ✓ | ✓ | ✓ | 4 |
| Prezio  2013 |  | ✓ | ✓ | ✓ | ✓ |  | 4 |
| Ramli  2016 | ✓ |  | ✓ | ✓ | ✓ |  | 4 |
| Schillinger 2009 |  |  | ✓ |  | ✓ |  | 4 (2 elements each for the two intervention groups) |
| Sonnichsen 2010 |  |  | ✓ | ✓ | ✓ | ✓ | 4 |
| Talavera  2021 |  |  | ✓ | ✓ | ✓ | ✓ | 4 |
| No. of Elements | 4 | 2 | 17 | 13 | 17 | 11 | 64 (Total number) |
