# Supplementary material for: Effectiveness of the chronic care model for adults with type 2 diabetes in primary care: a systematic review and meta-analysis
Source: Syst Rev. 2022 Dec 15;11:273. doi: 10.1186/s13643-022-02117-w (PMC9753411; doi:10.1186/s13643-022-02117-w)
Supplement: Supplementary file 7 — Additional file 7. Forest plot showing the effect of CCM on post-intervention LDL cholesterol (mmol/L). IV, inverse variance. [file 13643_2022_2117_MOESM7_ESM.docx]

Additional file 7: Forest plot showing the effect of CCM on post-intervention LDL cholesterol (mmol/L). IV, inverse variance

**
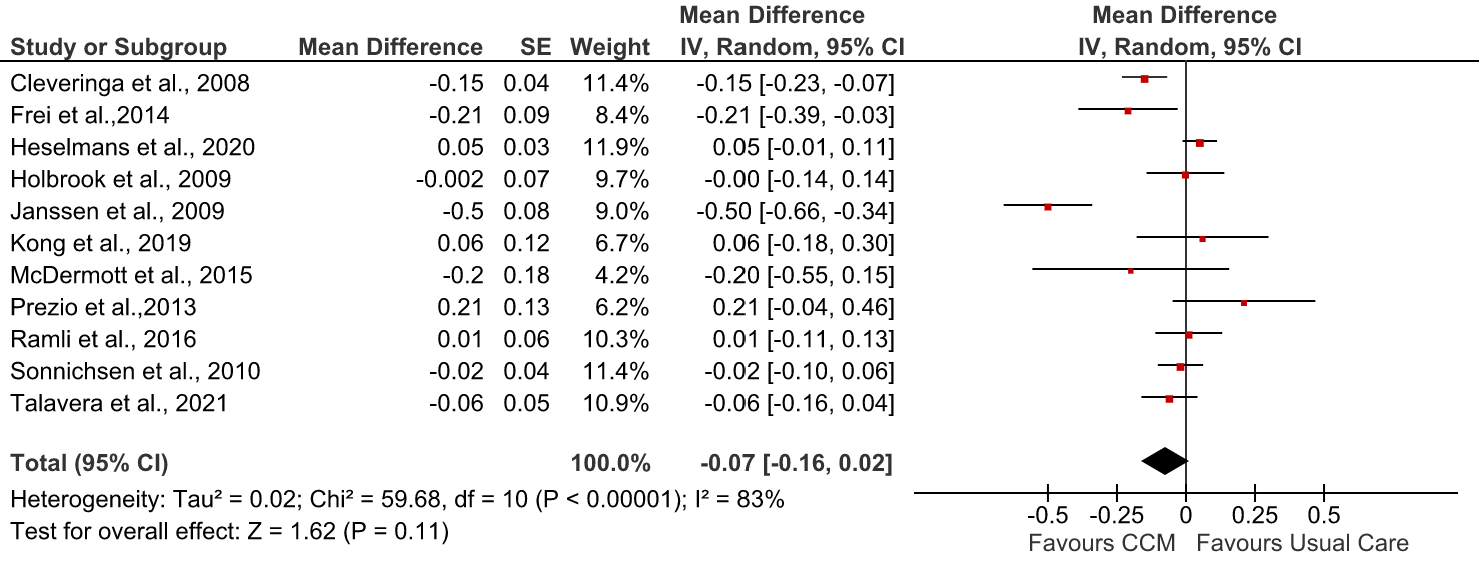
**
